# Success rate of single versus multiple debridement, antibiotics, and implant retention (DAIR) in hip and knee periprosthetic joint infection: a systematic review and meta-analysis

**DOI:** 10.1007/s00590-024-04091-6

**Published:** 2024-09-02

**Authors:** Loay A. Salman, Seif B. Altahtamouni, Harman Khatkar, Abdallah Al-Ani, Ghalib Ahmed

**Affiliations:** 1 Orthopedic Surgery Department, Hamad General Hospital, Hamad Medical Corporation, PO Box 3050, Doha, Qatar; 2 Royal London Hospital, Whitechapel, London, UK; 3 Office of Scientific Affairs and Research, King Hussein Cancer Center, Amman, Jordan; 4 Department of Orthopaedic Surgery, Surgical Specialty Center, Hamad Medical Corporation, Doha, Qatar

**Keywords:** Arthroplasty, Knee, Hip, Periprosthetic joint infection

## Abstract

**Purpose:**

This systematic review aimed to compare outcomes between multiple and single debridement, antibiotics, and implant retention (DAIR) procedures for early periprosthetic joint infection (PJI) in hip and knee arthroplasty.

**Methods:**

Four databases were searched from inception till January 2024 for original studies investigating the outcomes of multiple DAIR in hip and knee PJI. The primary outcome was the success rate in eradicating joint infection. This review was conducted per PRISMA guidelines.

**Results:**

A total of 9 observational studies with 1104 participants were included, with a mean age and BMI of 58.37 years (95%CI: 25.77–90.98) and 31.43 kg/m^2^ (95%CI: 28.89–34.98), respectively. The mean follow-up period was 58.37 months (95%CI: 25.77–90.98), and the average MINORS score assigned to the review was 17.6 ± 3.46, indicating a low overall risk of bias. An equivalent success rate between single and double DAIR was observed, at 67% (95%CI: 64–71%) and 70% (95%CI: 48–86%), respectively, with no statistically significant difference between the two treatment modalities (*p* = 0.740). Additionally, the success rate for triple DAIR ranged from 50 to 60%.

**Conclusion:**

This study suggests that double DAIR is a valid treatment option for acute PJI after TKA and THA, with a success rate comparable to single DAIR (70% vs. 67%, *p* = 0.740). Triple DAIR achieved success rates ranging from 50 to 60%. However, caution is warranted when interpreting these results due to heterogeneity in host comorbidity factors, DAIR protocols, and antibiotic regimens.

**Level of Evidence:**

Therapeutic, Level III.

**Supplementary Information:**

The online version contains supplementary material available at 10.1007/s00590-024-04091-6.

## Introduction

Hip and knee arthroplasty is one of the most common orthopedic procedures worldwide, with more than 2,244,587 primary and revision surgeries performed annually in the USA alone [1]. By 2030, the number of total knee arthroplasty (TKA) and total hip arthroplasty (THA) cases is projected to exceed 1.26 million and 500,000 annually, respectively [2–4].

Despite their generally high success rates, one of the most feared complications in knee and hip arthroplasty is periprosthetic joint infection (PJI). Approximately 1–2% of TKA and THA cases develop PJI within 2 years [5, 6], a figure expected to rise due to the aging population and the increasing number of joint replacements worldwide. PJI imposes a substantial burden on both patients and healthcare systems globally and can result in significant morbidity and necessitate revision surgeries [7, 8], leading to significant estimate costs, of around $50,000 to $100,000, particularly for infections involving antibiotic-resistant organisms [9, 10].

Diagnosing PJI can be challenging, yet the Musculoskeletal Infection Society (MSIS) criteria, established by Parvizi et al., have emerged as a standard reference for PJI diagnosis [11]. Treatment approaches for PJI vary depending on its type and chronicity, ranging from debridement, antibiotics, and implant retention (DAIR) to one-stage or two-stage component revision. Despite the inconsistent success rates reported in the literature, ranging from 33 to 88%, DAIR still emerges as an appealing treatment option for early acute PJI [12–17]. More recently, multiple serial DAIR procedures have been suggested as a valid treatment protocol in early PJI and found to be effective. However, small sample sizes with varying protocols have resulted in a setback [18, 19], and therefore, high-quality evidence is needed.

To the best of our knowledge, no previous systematic reviews have assessed the outcomes of double and multiple DAIRs. Therefore, the purpose of this study was to systematically review the effectiveness of multiple DAIRs and compare the success rate outcomes of single versus multiple DAIRs in treating early PJI in hip and knee arthroplasty. We hypothesize that there is no significant difference in success rates between patients treated with single DAIR versus those treated with double DAIR, and thus, double DAIR can be considered a valid treatment option when properly indicated.

## Methodology

This systematic review was conducted according to the preferred reporting items for systematic reviews and meta-analyses (PRISMA) guidelines [20]. The protocol was pre-registered on the international prospective register of systematic reviews (PROSPERO), registration number CRD42024513630.

### Search strategy

Four online databases (PubMed/Medline, Scopus, Embase, and Cochrane Library) were searched from inception to 25 January 2024 to identify all the studies investigating the outcomes of double or multiple DAIRs in patients with PJI following TKR and THR. The following keywords AND their derivative were included: “DAIR” OR “debridement” OR “antibiotic” OR “implant retention” AND “infection” AND “periprosthetic” AND “Arthroplasty” AND “hip” AND “knee.”

### Eligibility criteria

Studies were considered eligible if they satisfied the following criteria: (1) original RCTs or observational studies reporting the success rate of 1 or more DAIRs, (2) studies comparing the outcomes of multiple DAIRs to single DAIR, (3) DAIRs performed in both knee and hip acute PJIs (Up till 90 days), (4) no restriction on the type of implants or DAIR protocols included, (5) published in the English language. Exclusion criteria included: (1) studies not reporting the success rate of double or serial DAIRs, (2) studies reporting on chronic PJIs, (3) review articles, preclinical, cadaveric and anatomical studies, and case reports, (4) studies with incomplete or unextractable data for analysis, (5) Grey literature.

The main outcome of interest was the success rate of DAIR, defined as infection-free patient at follow-up, indicated by the absence of clinical signs suggestive of infection (fever, local pain, redness, warmth, sinus tract infection) and a negative c-reactive protein (CRP) level less than 10 mg/l [21, 22].

### Study screening

Two authors conducted the screening process independently and blindly by screening the titles and abstracts of the retrieved articles. For studies meeting the pre-specified eligibility criteria, a full-text review was performed. Any disagreement between the two authors was resolved by discussion with a more senior author. References of included articles were manually sought to ensure all relevant studies were included.

### Data extraction

Two authors independently extracted data from the included articles. The following data were collected: studies’ characteristics, patients’ demographics (such as age, sex, and body mass index), PJI criteria of diagnosis, Charlson comorbidity index, McPherson Host status, CCI Score, ASA Score, comorbidities, number of patients, number of knees, number of hips, number of DAIRs performed, number of single, double, and triple DAIRs, time between index surgery and frequent DAIRs, specific details of DAIR protocols performed, Type of infection (early acute or late hematogenous acute), type of micro-organism, detailed antibiotics regimen, overall complications, hospital stay, mean follow-up period, mortality, success, and failure rate.

### Quality assessment

The bias assessment in each study was conducted utilizing the methodological index for non-randomized studies (MINORS) criteria [23]. According to the MINORS criteria, comparative and non-comparative studies can achieve a maximum score of 24 and 16, respectively. Comparative studies are graded as very low quality (0–6), low quality (7–10), fair quality (11–16), good quality (16–20), and high quality (> = 20). Non-comparative studies are graded as very low quality (0–4), low quality (5–7), fair quality (8–12), and high quality (> = 13) [23]. Additionally, an Oxford Centre for Evidence-Based Medicine (OCEBM) [24] level of evidence (LoE) was determined for each study, followed by an overall GRADE recommendation for the entire review [25], in accordance with the Cochrane Collaboration Handbook.

### Quantitative assessment

The quantitative synthesis of data was conducted using R (version 4·0·2, R Core Team, Vienna, Austria, 2020) using the following packages and functions: “meta,” “metaprop,” “metamean,” “metagen,” “meta.reg,” and “forest.” The study outcomes were either expressed as pooled prevalence or raw means, both with their associated 95% confidence intervals. Heterogeneity among effect sizes was evaluated using *I*^2^ squared statistic. Definitions for heterogeneity were adapted from the Cochrane Handbook (> 25% mild, 25–50% moderate, > 50% severe). Due to the high *I*^2^ squared value (i.e., greater than 50%), a random effects model was utilized. Meta-regression for the included studies was technically possible but statistically meaningless due to having a low number of studies (less than 10) and even more modest sample sizes for some papers (e.g., 20).

## Results

### Search results

**Fig. 1:**
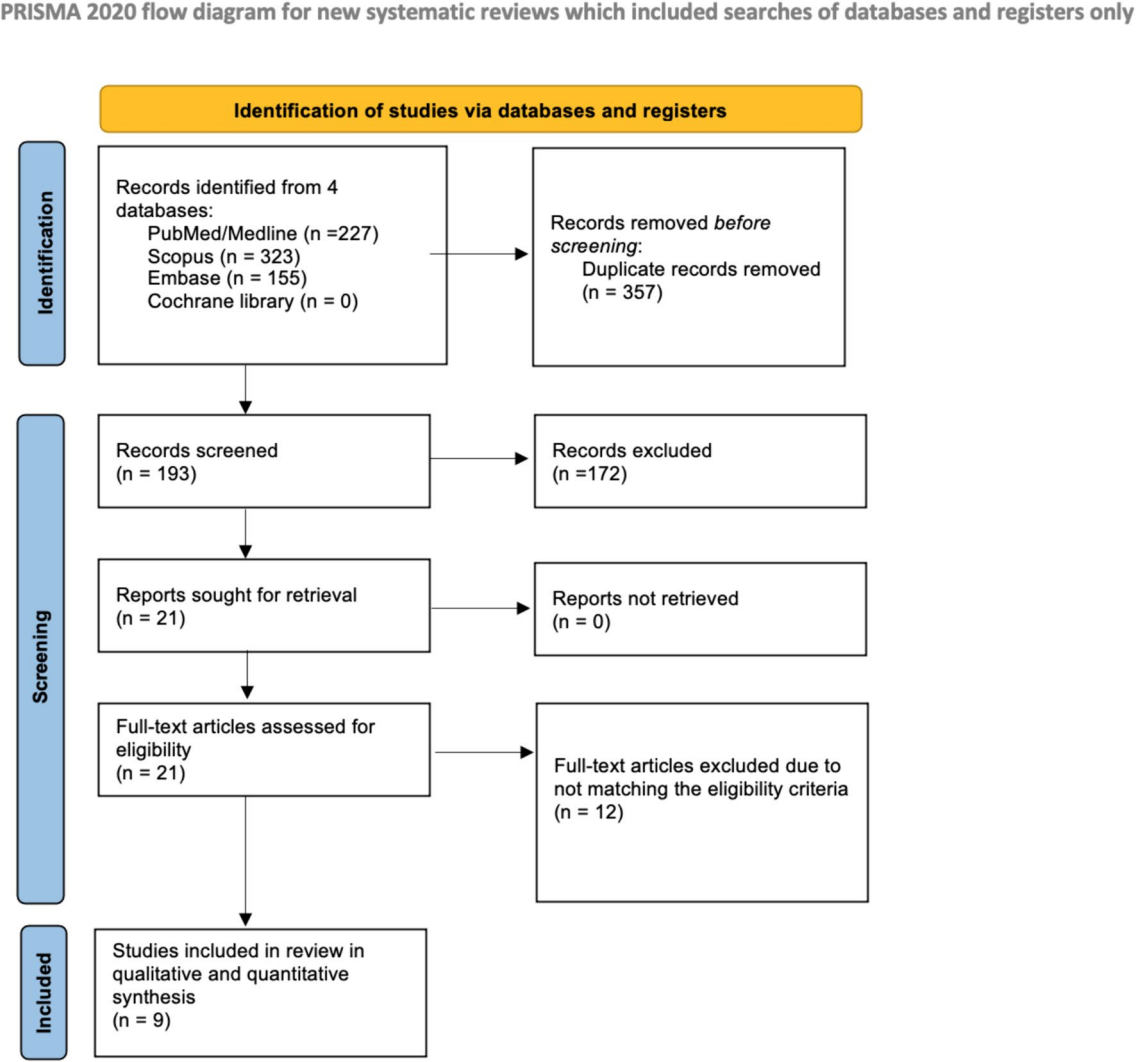
PRISMA flow diagram of record identification, screening, and selection in systematic review and meta-analysis

Rayyan AI website was used to manage the literature search results [26]. The initial database search returned 550 articles, from which 357 duplicates were eliminated, leaving 193 records for screening based on title and abstract. Among these, 172 were excluded, leaving 21 papers for full-text evaluation. Ultimately, nine studies [18, 19, 27–33] met the eligibility criteria and underwent qualitative and quantitative synthesis. The depiction of this process can be found in Fig. 1 of the PRISMA flowchart.

### Quality assessment (level of evidence and risk of bias)

Based on the OCEBM criteria, eight studies were level 3a, and one was level 2b, with an overall grade B of recommendation assigned to the review. This grading reflects the coherence of results among the studies included and suggests that this evidence is suitable for recommendation and general adherence in clinical practice [25]. The MINORS criteria scores of the included studies ranged from 11 to 22, with an average of 17.6 ± 3.46, indicating a low overall risk of bias. A summary of the quality assessment, according to the MINORS criteria, is presented in Table 1.

**Table 1:** MINORS criteria of the included studies

Study	Clearly stated aim	Inclusion of consecutive patients	Prospective data collection	Endpoints appropriate to study aim	Unbiased assessment of the study endpoint	Follow-up period appropriate to study aim	< 5% lost to follow-up	Prospective calculation of study size	Adequate control group	Contemporary groups	Baseline equivalence of groups	Adequate statistical analyses	Total
Chung (2019) [33]	2	2	2	2	0	2	1	2	–	–	–	–	13/16
Estes (2010) [19]	2	2	2	2	0	2	1	0	–	–	–	–	11/16
Mont (1997) [18]	1	2	2	2	0	2	2	0	2	2	1	1	17/24
Sancho (2022) [27]	2	2	2	2	0	2	2	2	2	2	2	2	20/24
Triantafyllopoulos (2015) [31]	2	1	1	2	0	2	2	2	1	2	2	2	19/24
Veerman (2022) [32]	2	2	1	2	0	2	1	2	1	2	1	2	18/24
Wouthuyzen-Bakker (2020) [30]	2	2	2	2	0	2	0	2	2	2	1	2	19/24
Grammatopoulos (2017) [28]	2	2	2	2	0	2	2	2	2	2	2	2	22/24
Perez (2022) [29]	2	1	1	2	0	2	2	2	2	2	1	2	19/24

### Studies characteristics

This meta-analysis included 9 studies with 1104 participants. The pooled mean age and BMI for the included studies were 58.37 years (95%CI; 25.77–90.98) and 31.43 kg/m^2^ (95%CI: 28.89–34.98), respectively. The pooled male-to-female ratio across studies was 1.23 (95%CI; 0.91–1.56). The mean pooled follow-up was 58.37 months (95%CI; 25.77–90.98). Table 2 summarizes the characteristics of the included studies.

**Table 2:** A summary of baseline study characteristics

Author (year)	Design, LoE	Country	Total patients	Age		Gender		BMI	
				Mean	SD	Male	Female	Mean	SD
Chung (2019) [33]	Retrospective, 3a	USA	83	Success: 68.1 Failure: 68.4	Success: 13.5 Failure: 15	43	40	Success: 30.9 Failure: 32.3	Success: 7.9 Failure: 8.4
Estes (2010) [19]	Retrospective, 3a	USA	20	67	NA	NA	NA	NA	NA
Mont (1997) [18]	prospective, 2b	USA	22	66.95	9.39	12	10	NA	NA
Sancho (2022) [27]	Retrospective, 3a	Spain	84	67.6	11.8	56	28	30.7	5.8
Triantafyllopoulos (2015) [31]	Retrospective, 3a	USA	141	Single DAIR: 64.8 Multiple DAIR: 62.2	Single DAIR: 10.7 Multiple DAIR: 12.6	69	72	Single DAIR: 31.4 Multiple DAIR: 33.3	Single DAIR: 7.6 Multiple DAIR: 12.8
Veerman (2022) [32]	Retrospective, 3a	Netherlands	88	66	11	48	40	30	6
Wouthuyzen-Bakker (2020) [30]	Retrospective, 3a	Netherlands	455	NA	NA	NA	NA	NA	NA
Grammatopoulos (2017) [28]	Retrospective, 3a	UK	122	71	NA	65	57	NA	NA
Perez (2022) [29]	Retrospective, 3a	USA	89	64	NA	46	43	Single DAIR: 35.79 Double DAIR: 35.31	NA

Author (year)	PJI type		Knees			Hips			Follow-ups (months)	
	Early/postsurgical	Late/hematogenous	Total (*n*)	Primary (*n*)	Revisions (*n*)	Total (*n*)	Primary (*n*)	Revisions (*n*)	Mean	SD
Chung (2019) [33]	23	60	48	31	17	35	17	18	Success: 42.7 Failure: 35.7	Success: 35.2 Failure: 26.6
Estes (2010) [19]	2	18	16	NA	NA	4	NA	NA	42	NA
Mont (1997) [18]	10	14	24	18	6	0	0	0	45.12	25.6
Sancho (2022) [27]	72	12	37	35	2	47	32	15	67.4	33.6
Triantafyllopoulos (2015) [31]	71	70	81	NA	NA	60	NA	NA	Single DAIR: 64.1 Multiple DAIR: 75.2	Single DAIR: 46.6 Multiple DAIR: 53.6
Veerman (2022) [32]	NA	NA	35	0	35	53	0	53	NA	NA
Wouthuyzen-Bakker (2020) [30]	Second DAIR: 129	Second DAIR: 15	121	NA	NA	334	NA	NA	NA	NA
Grammatopoulos (2017) [28]	74	48	0	0	0	122	82	40	84	NA
Perez (2022) [29]	22	67	64	32	32	25	14	11	Single DAIR: 25.1 Double DAIR: 15.1	NA

LoE, level of evidence; DAIR, debridement, antibiotics, and implant retention; BMI, body mass index; PJI, periprosthetic joint infection; SD, standard deviation; and NA, not available

### Baseline host health status and comorbidities

Across 8 included studies comprised of 1016 participants, 41% (95%CI; 22–63%) of participants had early PJI. On the other hand, 47% (95%CI; 18–78%) had late PJIs. Primary knee and hip arthroplasties had pooled prevalence rates of 74% and 63% among 173 and 229 participants, respectively. Among 208 and 282 participants, the pooled prevalences of revision knee and hip arthroplasties were 50% and 64%, respectively. Veerman et al. reported that all participants within their cohort had knee and hip revisions. The baseline comorbidities of the included patients are shown in Table 3.

**Table 3:** A summary of baseline patient comorbidity profiles

Author (year)	McPherson Host status									CCI score			ASA Score			
	Infection type			Host type			Extent of infection			Mean	SD	Range	I	II	III	IV
	I	II	III	A	B	C	1	2	3							
Chung (2019) [33]	24	59	0	28	37	18	NA	NA	NA	Success: 2.5 Failure: 3.6	Success: 1.9 Failure: 2.9	NA	NA	NA	NA	NA
Estes (2010) [19]	2	18	0	8	8	4	16	4	0	NA	NA	NA	NA	NA	NA	NA
Mont (1997) [18]	NA	NA	NA	NA	NA	NA	NA	NA	NA	NA	NA	NA	NA	NA	NA	NA
Sancho (2022) [27]	NA	NA	NA	NA	NA	NA	NA	NA	NA	NA	NA	0–3: 32, 4 or more: 52	9	43	30	2
Triantafyllopoulos (2015) [31]	NA	NA	NA	NA	NA	NA	NA	NA	NA	NA	NA	NA	0	87	54	0
Veerman (2022) [32]	NA	NA	NA	NA	NA	NA	NA	NA	NA	NA	NA	NA	NA	NA	NA	NA
Wouthuyzen-Bakker (2020) [30]	NA	NA	NA	NA	NA	NA	NA	NA	NA	NA	NA	NA	NA	NA	NA	NA
Grammatopoulos (2017) [28]	NA	NA	NA	NA	NA	NA	NA	NA	NA	1	NA	0–8	16	55	43	8
Perez (2022) [29]	22	67	0	26	38	25	43	45	1	Single DAIR: 3.76 Double DAIR: 3.88	NA	NA	0	18	62	9

Author (year)	Comorbidities (*n*)							
	Diabetes	Inflammatory arthropathy	Liver disease	Renal disease	Cardiac disease	Immunosuppression (state and/or drugs)	Smoking	alcohol
Chung (2019) [33]	10	6	NA	1	NA	5	NA	NA
Estes (2010) [19]	2	1	NA	2	NA	8	NA	2
Mont (1997) [18]	4	2	NA	NA	NA	2	NA	NA
Sancho (2022) [27]	19	NA	3	9	26	6	40	17
Triantafyllopoulos (2015) [31]	26	NA	7	28	73	NA	20	NA
Veerman (2022) [32]	11	12	NA	NA	NA	8	NA	NA
Wouthuyzen-Bakker (2020) [30]	31	13	NA	7	55	22	24	NA
Grammatopoulos (2017) [28]	16	NA	NA	4	5	NA	NA	NA
Perez (2022) [29]	NA	NA	NA	NA	NA	NA	NA	NA

CCI, Charlson comorbidity index; ASA, American Society of Anesthesiologists Classification

Across 4 studies reporting on ASA types (Table 3), two reported no ASA type I risk among their combined cohorts of 263 participants. Sancho et al. and Gramamtopoulos et al. reported ASA type I risk rates of 11% and 13%, respectively. The pooled prevalence of ASA type II risk across 436 participants was 44% (95%CI; 21–70%), while ASA type III risk had a pooled prevalence of 45% (95%CI; 24–67%). Triantafyllopoulos et al. reported zero prevalence of ASA type IV risk among their cohort of 141 participants. On the other hand, three cohorts, comprised of 295 participants, reported a pooled prevalence of 3% (95%CI; 0–23%) of ASA type IV risk (Table 3).

### Success rate of DAIR

**Fig. 2:**
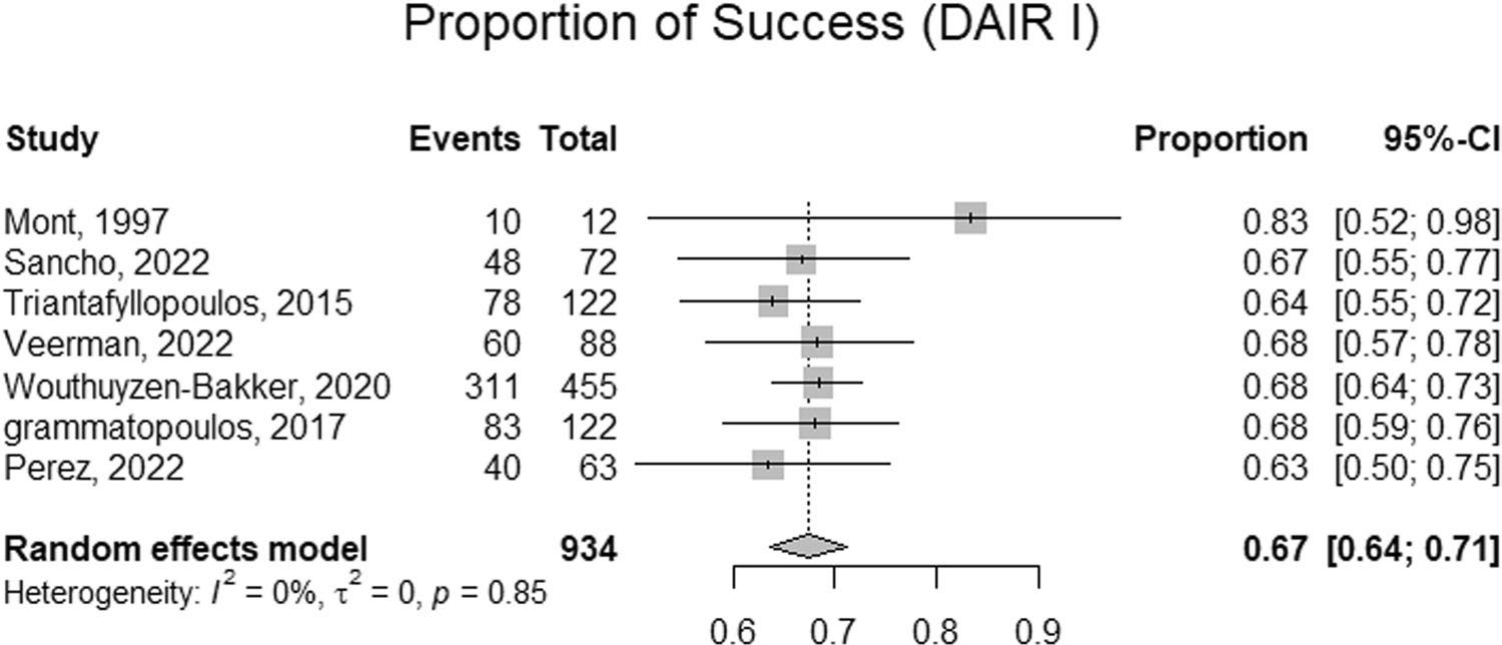
A forest plot demonstrating the pooled success rate of single DAIR procedures

**Fig. 3:**
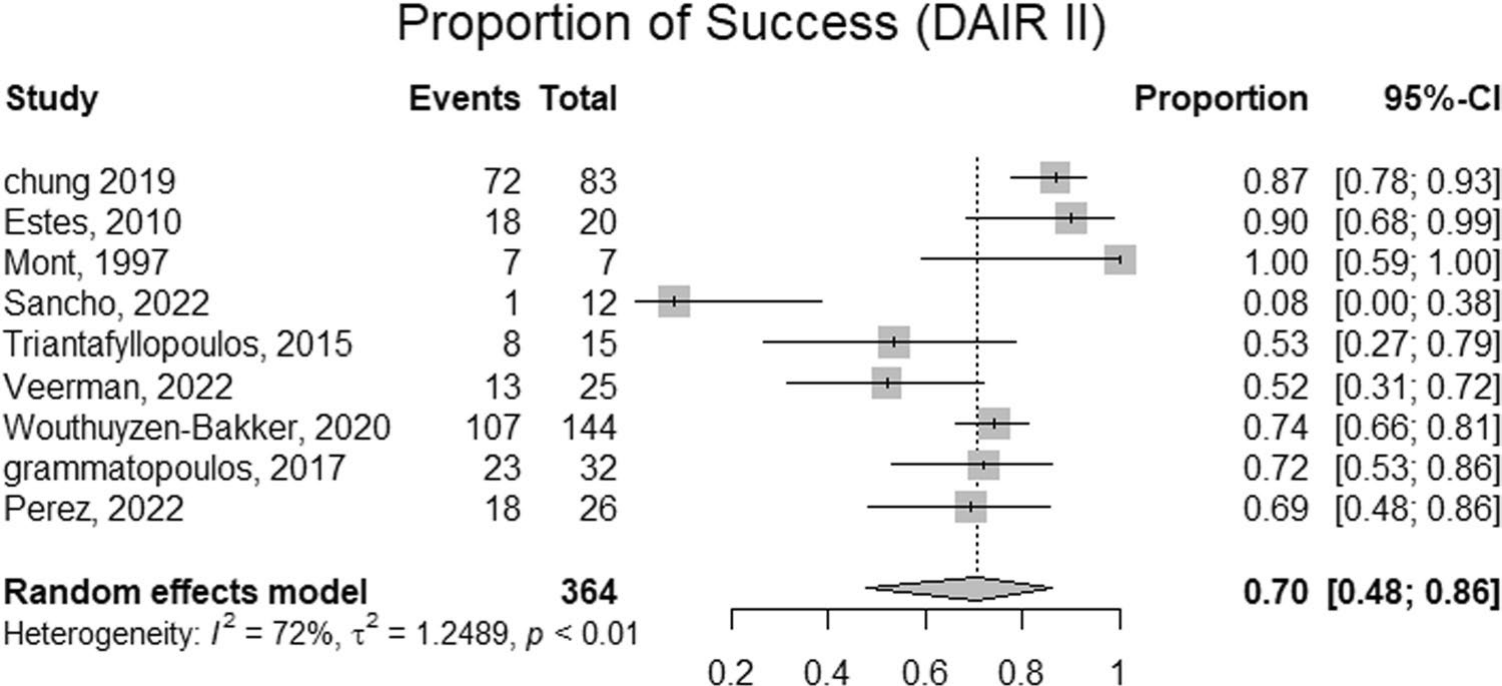
A forest plot demonstrating the pooled success rate of single DAIR procedures

**Fig. 4:**
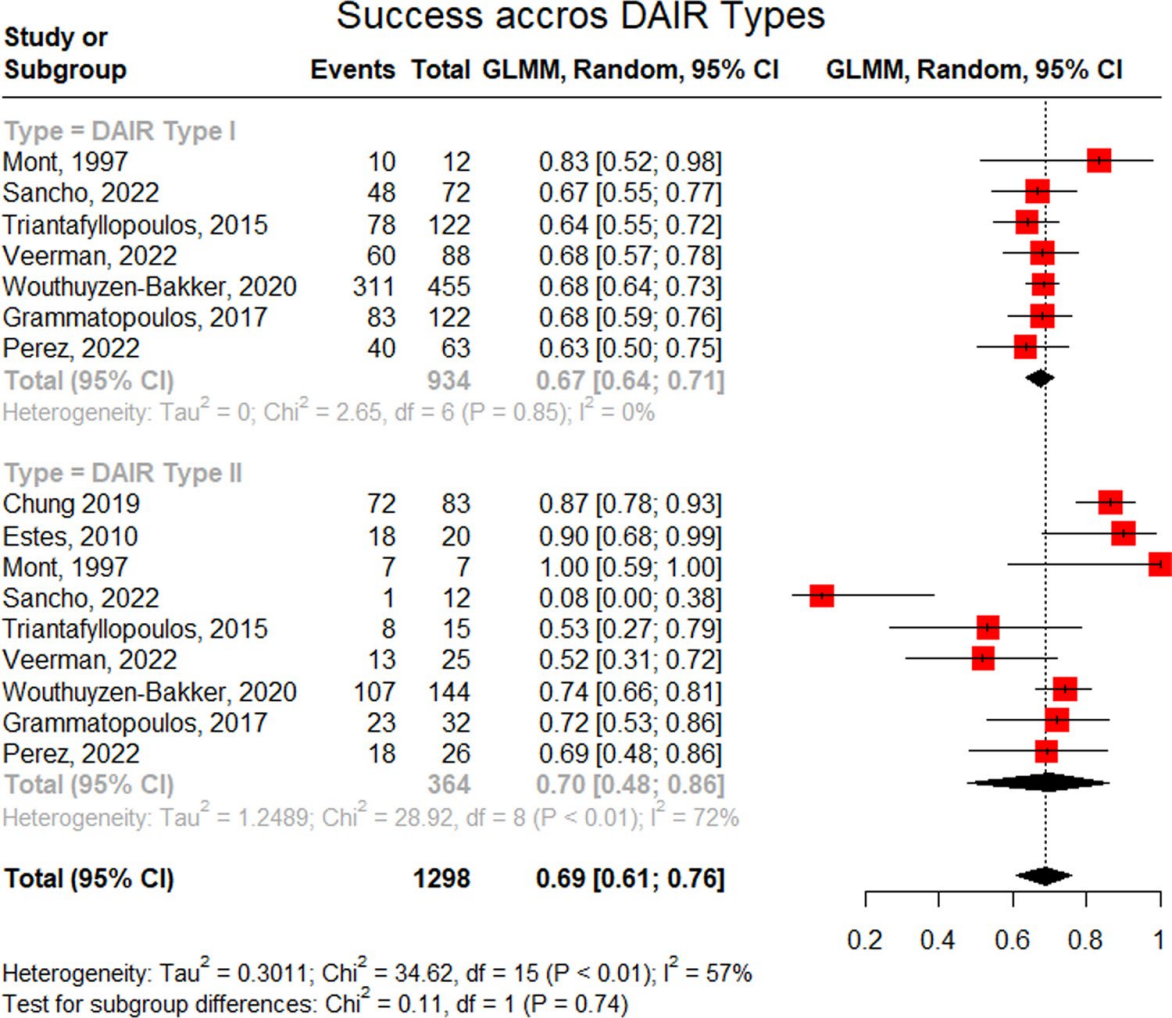
Forest plot comparison of single and double DAIR

**Fig. 5:**
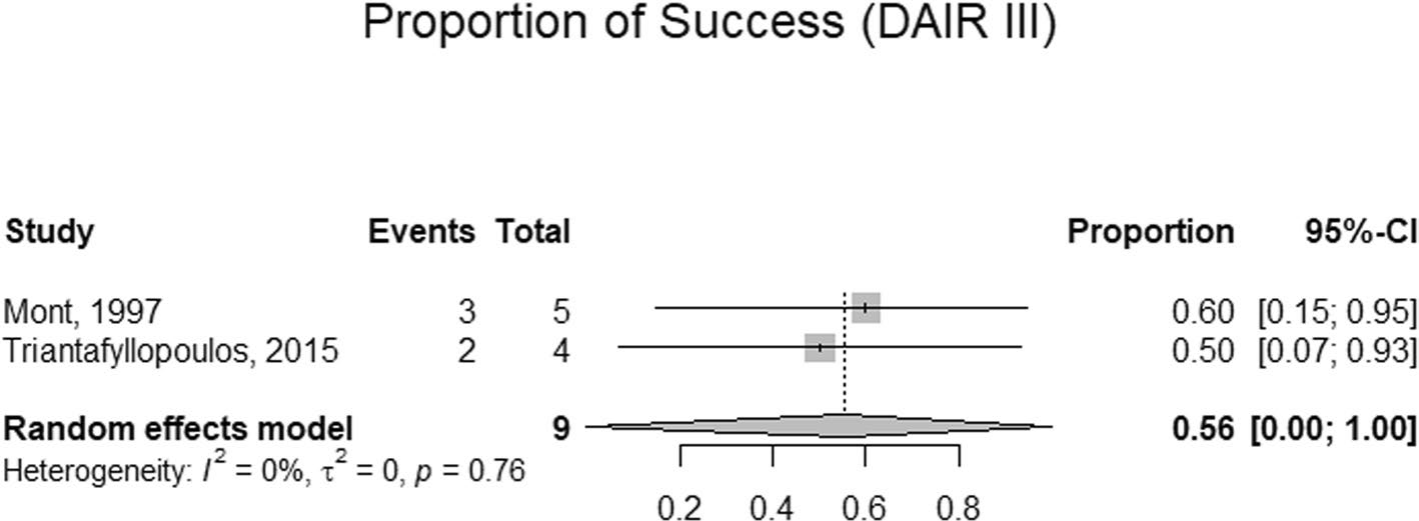
A forest plot demonstrating the pooled success rate of Triple DAIR procedures

Three studies included participants with exclusive usage of a single DAIR. Across included studies, the pooled prevalence of a single DAIR was 98%. Similarly, two studies exclusively utilized (or reported on) exclusive usage of double DAIR. Across these reports, the proportion of double DAIR was 53%. Mont et al. and Triantafyllopoulos et al. reported usage rates of triple DAIR corresponding to 23% and 3%, respectively [refer to Figs. 2, 3, 4 and 5 and Supplementary material]. The success rate of a single DAIR was 67% (95%CI; 64–71%) among 934 participants (Fig. 2). Similarly, the success rate of double DAIR was 70% (95%CI; 48–86%) among a pooled cohort of 364 participants (Fig. 3). The success rates between single and double DAIR were not statistically significant (*p* = 0.740) (Fig. 4). Finally, the success rate for triple DAIR ranged from 50 to 60%, as reported by Mont et al. and Triantafyllopoulos et al., respectively (Fig. 5). Regression analysis demonstrated that sample size was an independent effector of success rate in single and double DAIR procedures among included studies (*p* = 0.0012 and 0.0068, respectively). Furthermore, the detailed DAIR procedure protocols, microbiological profiles, and antibiotic regimens used in each included study are summarized in Tables 4 and 5, respectively.

**Table 4:** A qualitative summary of DAIR procedure details

Author (year)	PJI diagnostic criteria used	No. of DAIRs	Duration between 1st and 2nd DAIR		
			Mean	SD	Range
Chung (2019) [33]	MSIS	2	Success: 5.3 Failure: 5.7	Success: 1.7 Failure: 2.6	NA
Estes (2010) [19]	MSIS	2	NA	NA	3–7
Mont (1997) [18]	Infection was diagnosed by aspiration of the knee before operative debridement	3	NA	NA	NA
Sancho (2022) [27]	EBJIS definition	2	31.8	29.4	NA
Triantafyllopoulos (2015) [31]	MSIS	3	61.4	137.6	NA
Veerman (2022) [32]	Based on the typical signs of inflammation, a fistula, or persistent wound drainage (15–10 days postoperatively)	2	NA	NA	NA
Wouthuyzen-Bakker (2020) [30]	MSIS	2	NA	NA	Within 28
Grammatopoulos (2017) [28]	MSIS	2	NA	NA	NA
Perez (2022) [29]	MSIS	2	16	NA	9–36

Author (year)	Incision opening	Synovectomy	Infected soft tissue debridement	Modular parts exchange	Implants irrigation
	Pre-existing	Taking cultures	Taking cultures	(yes/no/NA)	Saline, betadine
	(yes/no/NA)	(yes/no/NA)	(yes/no/NA)		(yes/no/NA)
Chung (2019) [33]	NA	Yes	Yes	Yes	Yes
Estes (2010) [19]	NA	Yes	Yes	Yes	Yes
Mont (1997) [18]	Yes	Yes	Yes	Yes except 3 patients	Yes
Sancho (2022) [27]	Yes	Yes	Yes	Only 4 patients	Yes
Triantafyllopoulos (2015) [31]	Yes	Yes	Yes	Yes	Yes
Veerman (2022) [32]	Yes	Yes	Yes	Yes	Yes
Wouthuyzen-Bakker (2020) [30]	NA	Yes	Yes	Yes	Yes
Grammatopoulos (2017) [28]	NA	Yes	Yes	65 patients	Yes
Perez (2022) [29]	NA	Yes	Yes	Yes	Yes

MSIS, Musculoskeletal Infection Society; EBJIS, European Bone and Joint Infection Society

**Table 5:** A qualitative summary of the microbiological profile and culture-specific antibiotics

Author (year)	Organisms						Post-DAIR culture-specific antibiotics		
	Most common		2nd most common		3rd most common		Regimen	Duration (weeks)	
	1st DAIR	2nd DAIR	1st DAIR	2nd DAIR	1st DAIR	2nd DAIR		IV	Oral
Chung (2019) [33]	NA	*MSSA*	NA	*MRSA*	NA	Polymicrobial	Rifampin combination therapy	6	NA
Estes (2010) [19]	*MSSA*	*MRSA*	*MSSA*	NA	*Strep. Pneumonia*	NA	Rifampin combination therapy	6	9
Mont (1997) [18]	*Staph. aureus*	NA	*Staph. epidermidis*	NA	*B-hemolytic strep*	NA	NA	6–12	NA
Sancho (2022) [27]	*CoNS*	*MSSA*	*MSSA*	Polymicrobial	Polymicrobial	*CoNS/Strep. dysgalactiae/Staph. lugdunensis/E. faecalis/Serratia marcescens*	Rifampin combination therapy	1–2	6–12
Triantafyllopoulos (2015) [31]	*MSSA*	*MSSA*	*MRSA*	*MRSA*	Other gram-positive bacteria	*E. faecalis/E. cloacae*	NA	6	6
Veerman (2022) [32]	*Staphylococcus spp.*	NA	*Gram-negative bacilli*	NA	*Streptococcus spp.*	NA	Rifampin combination therapy	13	
Wouthuyzen-Bakker (2020) [30]	*Staph. aureus*	NA	Polymicrobial	NA	*Streptococci*	NA	Rifampin combination therapy	At least 2	10
Grammatopoulos (2017) [28]	*Staph. aureus*	NA	Polymicrobial	NA	*CoNS*	NA	Ceftriaxone/teicoplanin, Ciprofloxacin, rifampicin	NA	6
Perez (2022) [29]	*MSSA/MRSA*	*MSSA/MRSA*	Polymicrobial	*Group B Strep*	*Group B strep*	Polymicrobial	NA	6	13

MSSA, methicillin-susceptible *Staphylococcus aureus*; MRSA, methicillin-resistant *Staphylococcus aureus*; and CoNS, coagulase-negative* staphylococci*

## Discussion

The clinical evidence for the efficacy of DAIR in acute periprosthetic joint infection is compelling; however, the utility of repeated debridements has not been fully evaluated [34–37]. This is the first systematic review in the literature to compare the effectiveness of single DAIR to double DAIR and provide evidence-based recommendations for treating clinicians.

The main finding of this analysis is that both single and double DAIR techniques, when correctly indicated, are effective treatments for early acute PJI after TKA and THA, with comparable success rates of 67% and 70%, respectively (*p* = 0.740). This contrasts with the latest International Consensus Meeting on PJI, which does not support serial debridements for infected hip and knee replacements and recommends component removal after an unsuccessful initial debridement [38–41].

The proposed mechanisms to explain this finding relate to surgical, implant, and patient factors surrounding serial debridements [27]. One such mechanism could relate to the idea that if an initial DAIR is unsuccessful, this could, in part, be due to an underlying host with a significant medical background allied with a more complex microbiological profile—making any initial or serial debridements more challenging both surgically and medically—thus decreasing the overall rate of success of any subsequent debridement. Similarly, a further surgical procedure to eradicate infection may conversely introduce iatrogenic infection, thus diminishing overall success rates of two- or three-stage debridements.

The concept of antibiotic resistance in PJI is well established within the microbiology and orthopedic literature [42, 43]. Theoretically, if a debridement alongside a course of antibiotics is clinically unsuccessful, subsequent prolonged antimicrobial administration may promote pathogenic-mediated antimicrobial resistance and thus reduce the chances of a successful second debridement. Careful liaison with infectious disease colleagues is required if a second DAIR is being considered—to ensure both appropriate microbiological agent choice alongside finding a suitable course length.

Further, one key consideration in the success of repeat DAIR is the quality of the periarticular soft tissue envelope [44, 45]. After an unsuccessful first DAIR, soft tissue defects, such as a fistula or draining sinus, will dramatically increase the technical difficulty of a subsequent surgical debridement. Particularly in the field of revision knee arthroplasty, where soft tissue envelopes can be fragile even in a primary arthroplasty procedure, repeated debridements will likely further destabilize the surrounding tissues and thus could be a possible cause for clinical failure of repeated DAIR [44, 46, 47].

This analysis reports that the utilization of triple DAIR, considered the most radical surgical technique, does not confer a higher clinical success rate than single or double DAIR. Biological factors such as biofilm formation after repeated debridements and diminished host surgical fitness throughout multiple surgical procedures should be considered within the context of this finding [18, 48]. Similarly, the timing of repeated debridements, for instance, whether repeated debridements are in close proximity or after a significant time period, needs to be further evaluated. The diminishing rate of success after serial debridements brings into focus the utility of single-, one-, and two-stage revision arthroplasty as tools to definitively eradicate periprosthetic joint infection [49–51].

## Limitations

Within the context of this analysis, the included studies have homogenized both hip and knee DAIR as one surgical entity, disregarding the surgical contrast between DAIR in knee and hip arthroplasty surgery. Technical considerations relating to this should be considered—for instance, in hip arthroplasty DAIR—the prosthesis is dislocated to allow for modular component exchange, often with a violation of the musculature that surrounds the hip [52, 53]. The associated morbidity associated with this manoeuver, in comparison with DAIR for TKR, furthers the notion that DAIR is a joint-specific entity, with further analyses sub-categorizing knee and hip DAIR as separate cohorts.

It is also worth considering that the studies included in this analysis varied in their selection criteria for choosing single or staged debridements in specific patients. Treating clinicians may have opted to employ a two- or three-stage strategy in patients with more challenging comorbidities or more complex microbiological profiles and conversely opted for a single debridement strategy in a more physiologically robust patient with a more treatable pathogen. This element of bias has not been accounted for within this analysis due to the limited sample size and inconsistent reporting of some studies, which prevent meaningful regression analysis. Accounting for this discrepancy should form the basis of any future high-quality prospective research that seeks to examine this question more robustly.

## Conclusion

This study demonstrates that double DAIR, when properly indicated, is a valid treatment for acute PJI after TKA and THA, with success rates similar to single DAIR (70% and 67%, respectively, *p* = 0.740). Triple DAIR had a success rate of 50–60%. However, results should be interpreted cautiously due to differences in host comorbidities, DAIR protocols, and antibiotic regimens. Future large-scale studies should address these confounders.

## Supplementary Information

Below is the link to the electronic supplementary material.

Supplementary file1 (DOCX 38328 KB)

## Data Availability

Available upon request.
