# Supplementary figures and images for: Success rate of single versus multiple debridement, antibiotics, and implant retention (DAIR) in hip and knee periprosthetic joint infection: a systematic review and meta-analysis
Source: Eur J Orthop Surg Traumatol. 2024 Sep 2;34(8):3859–72. doi: 10.1007/s00590-024-04091-6 (PMC11519117; doi:10.1007/s00590-024-04091-6)

**Appendix.**

**Supplementary Material.**


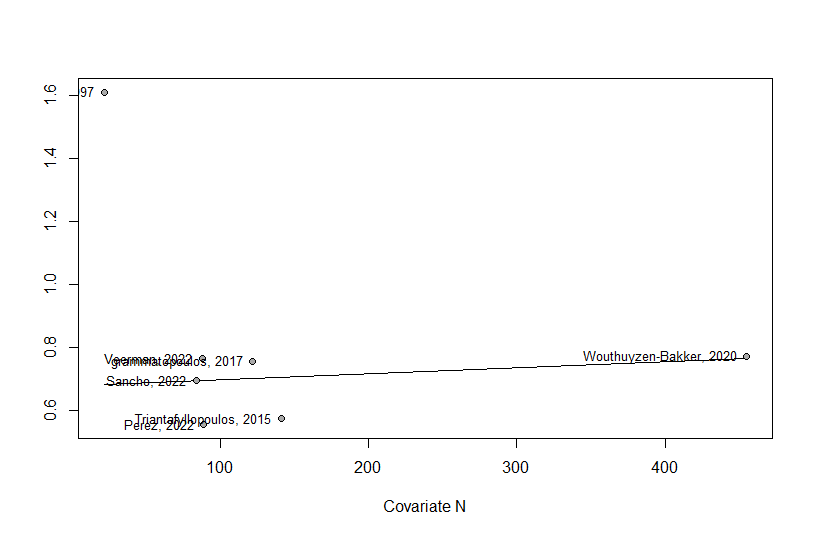


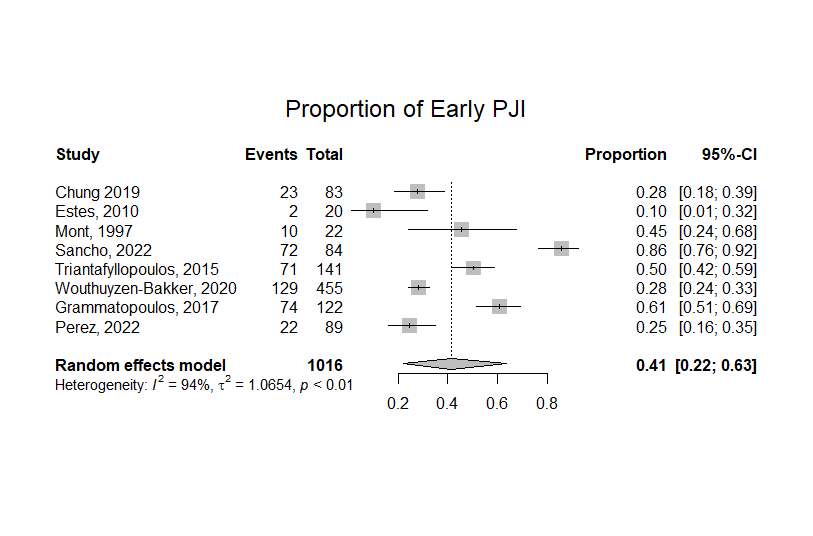

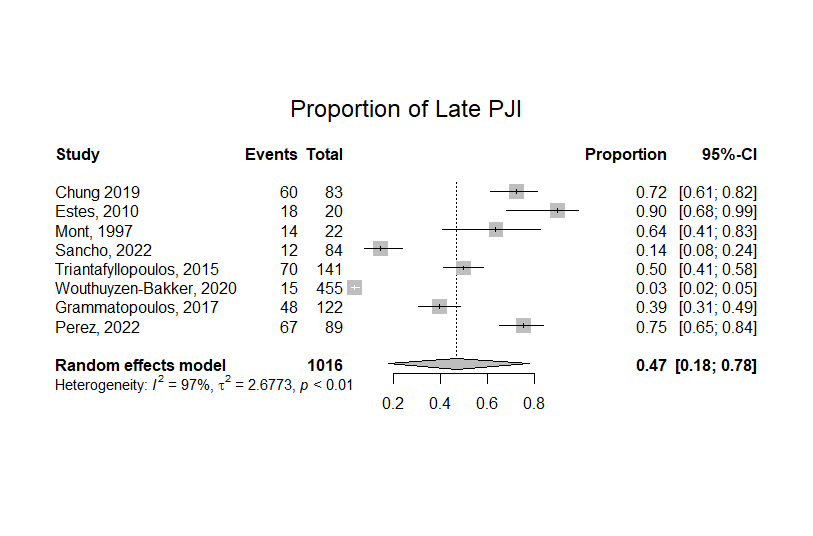

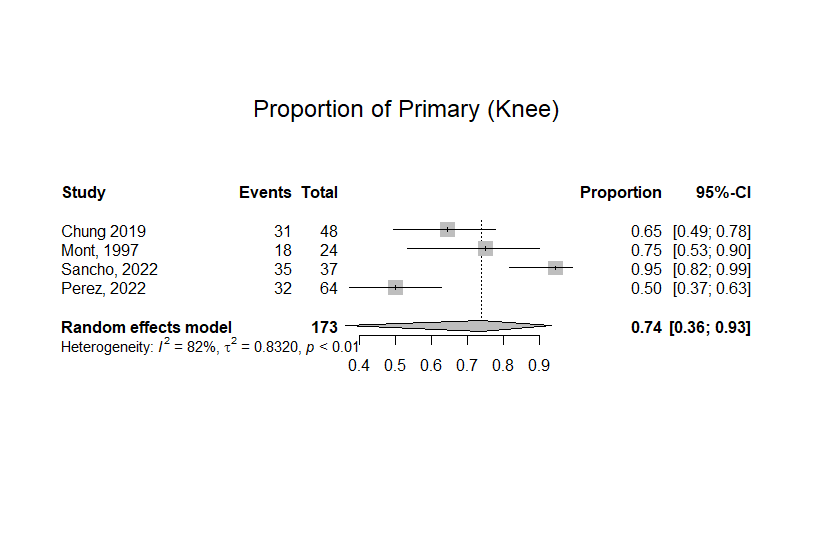

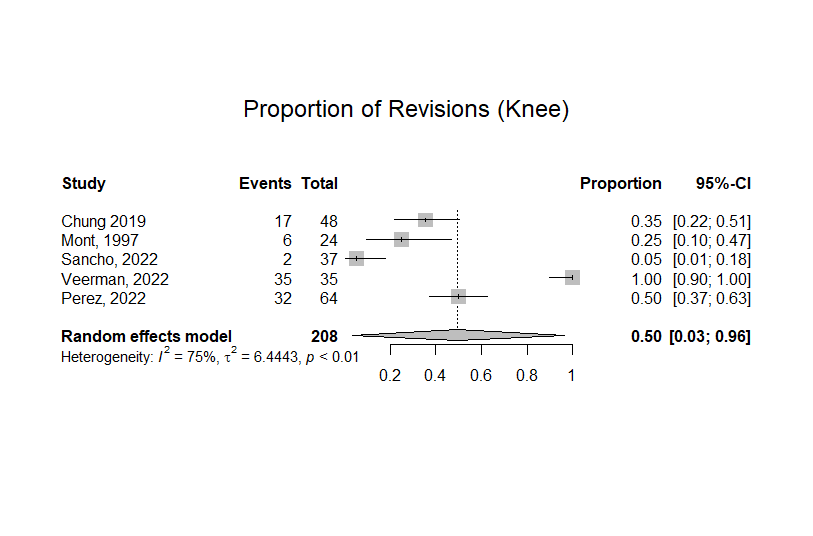

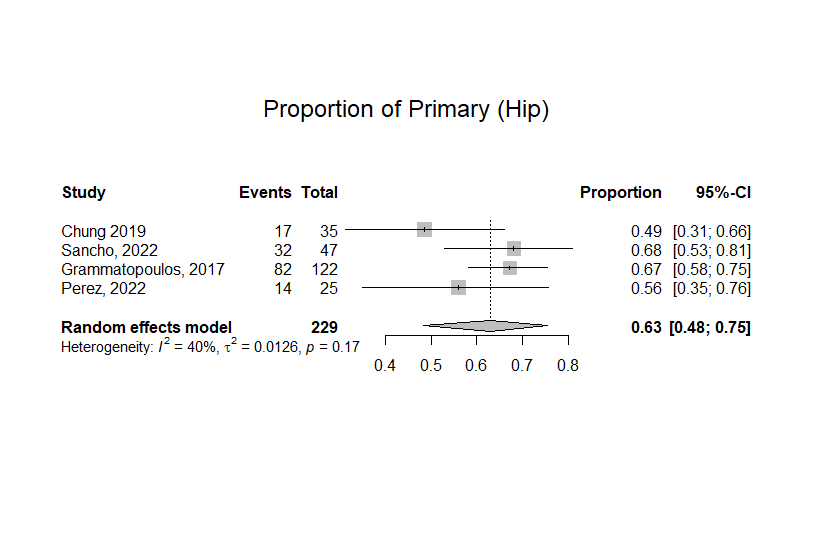

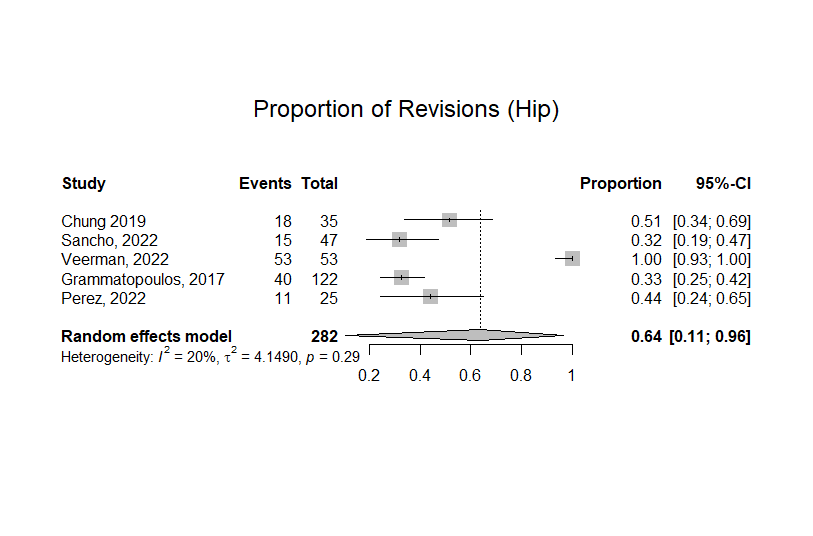

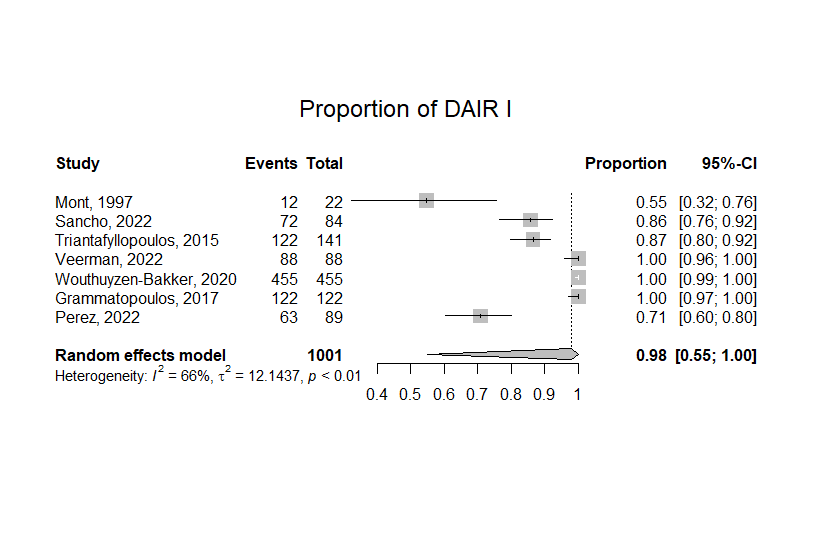

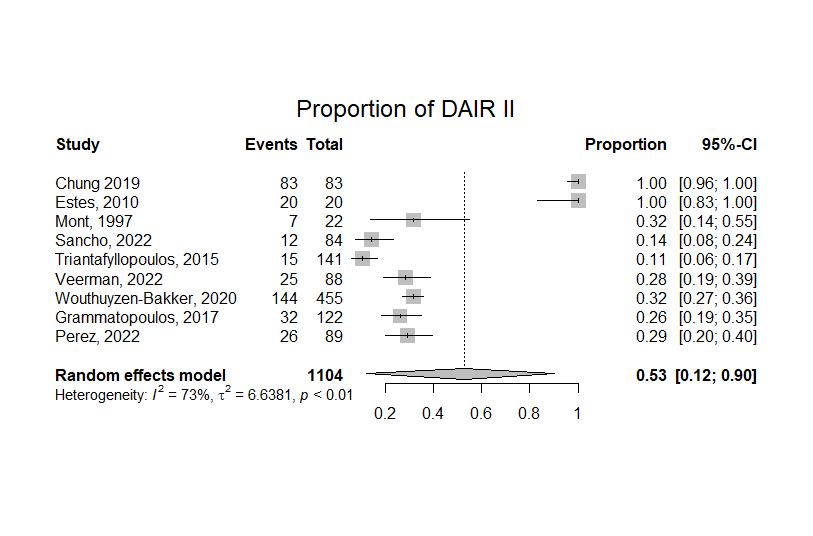

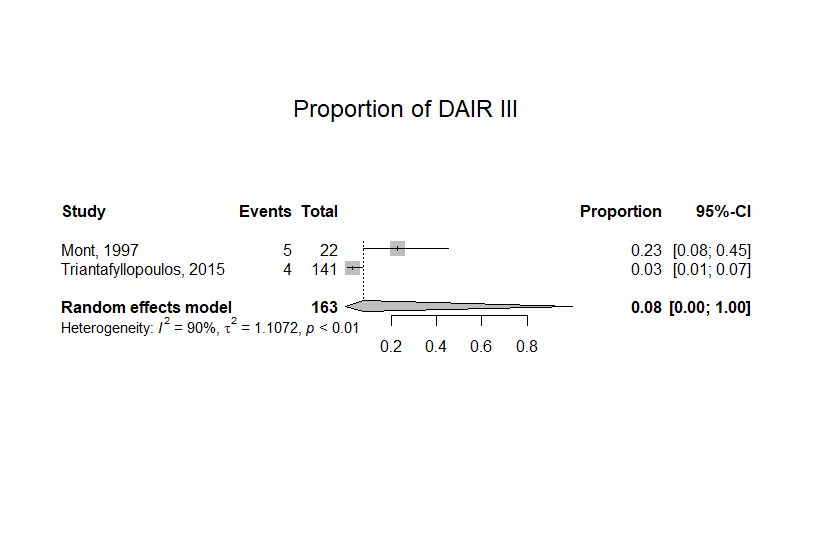

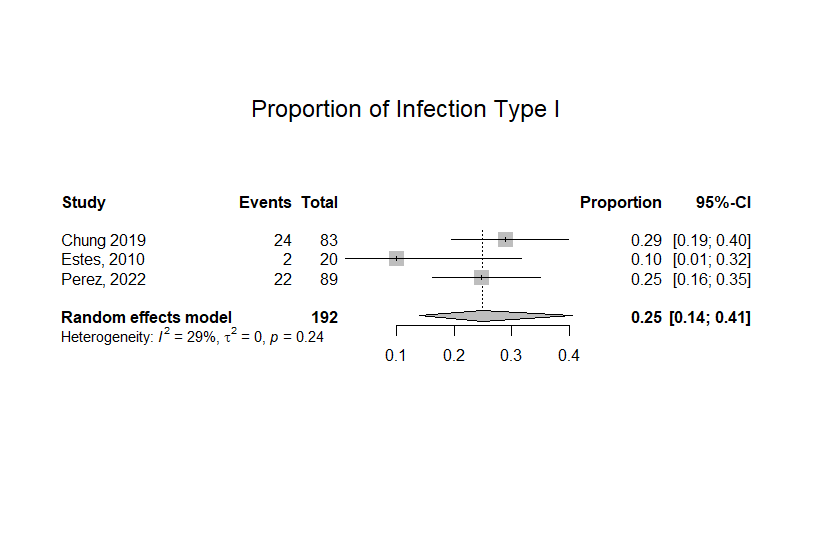

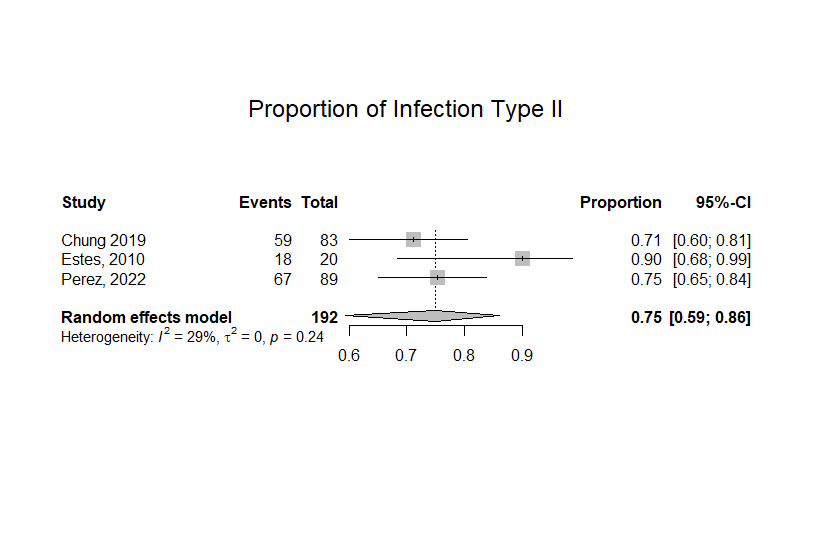

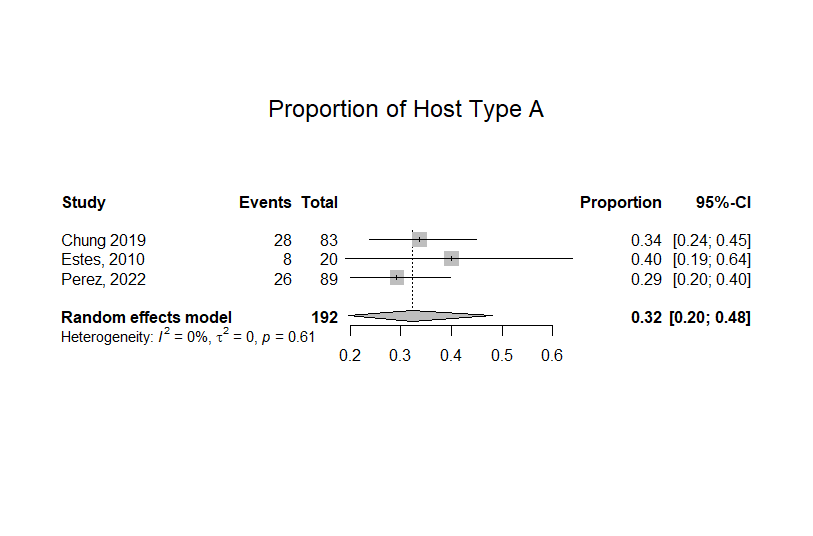

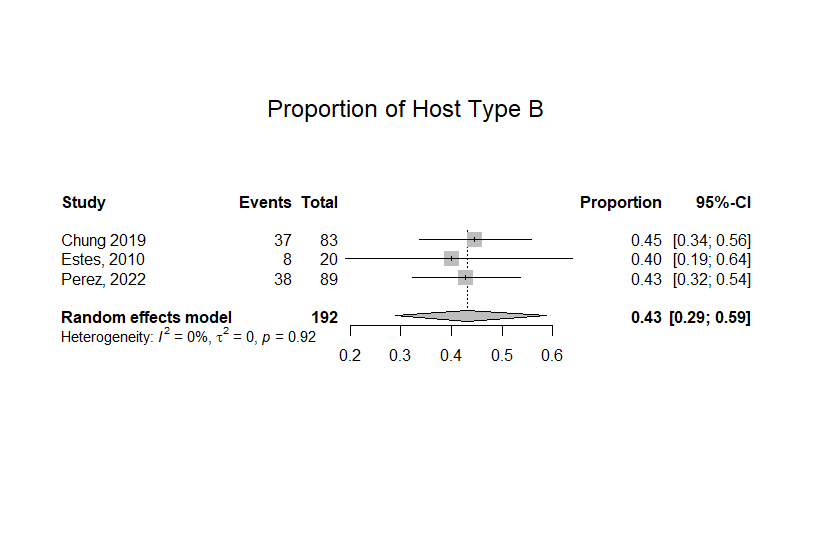

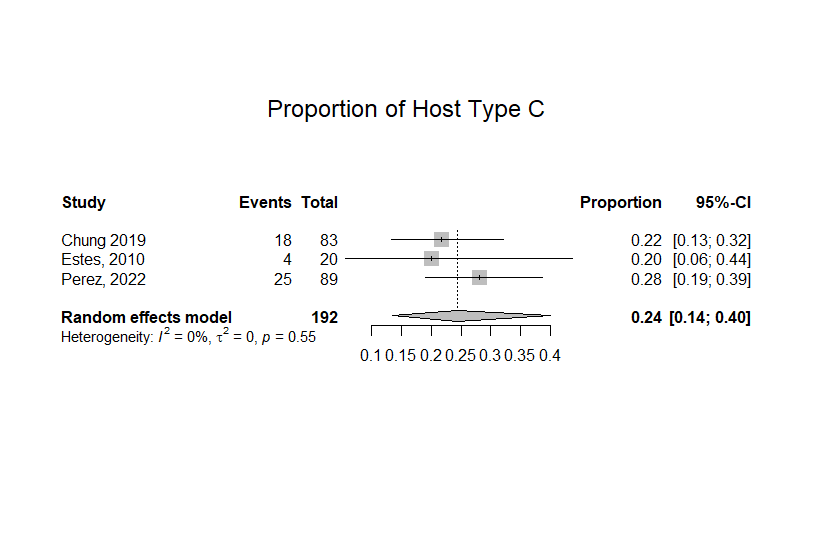

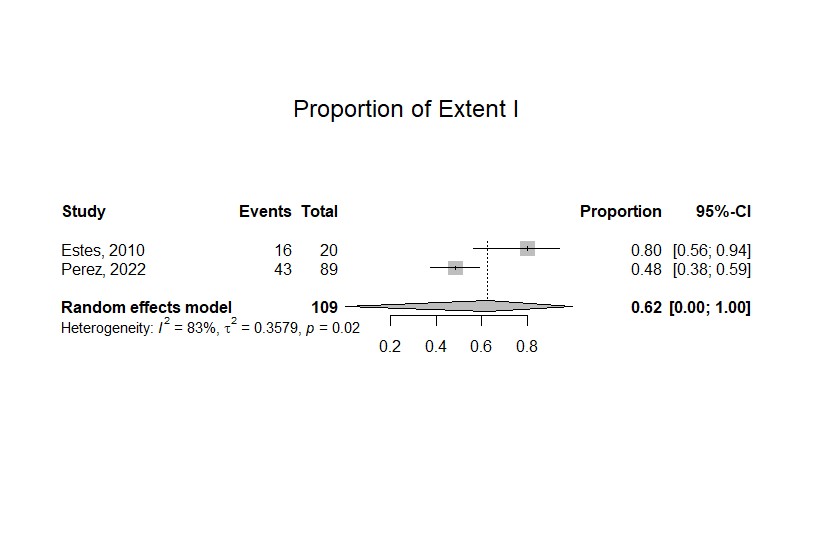

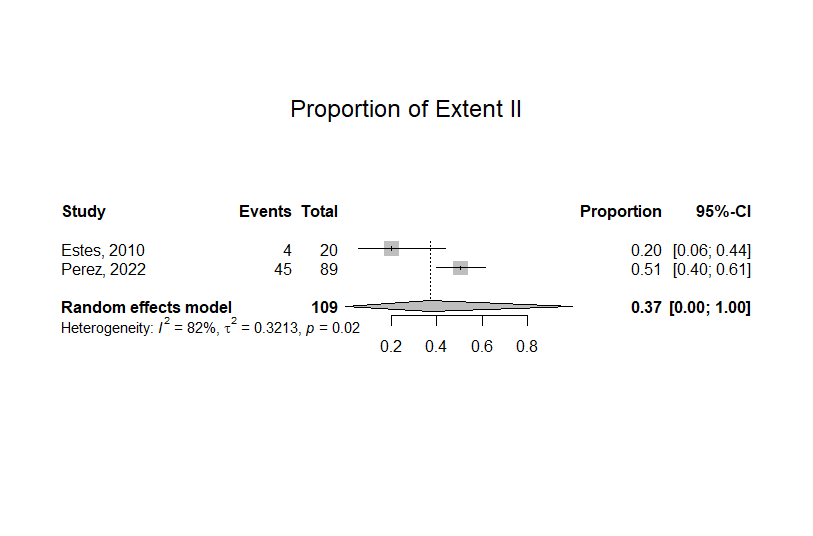

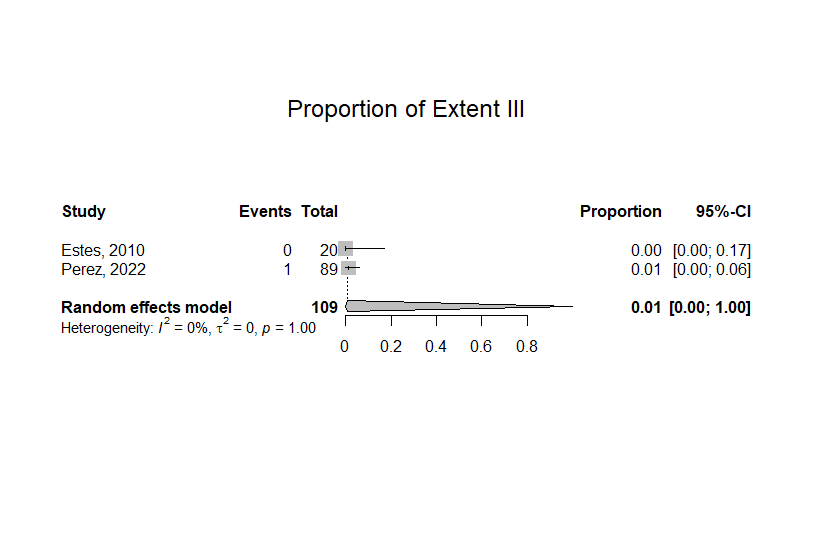

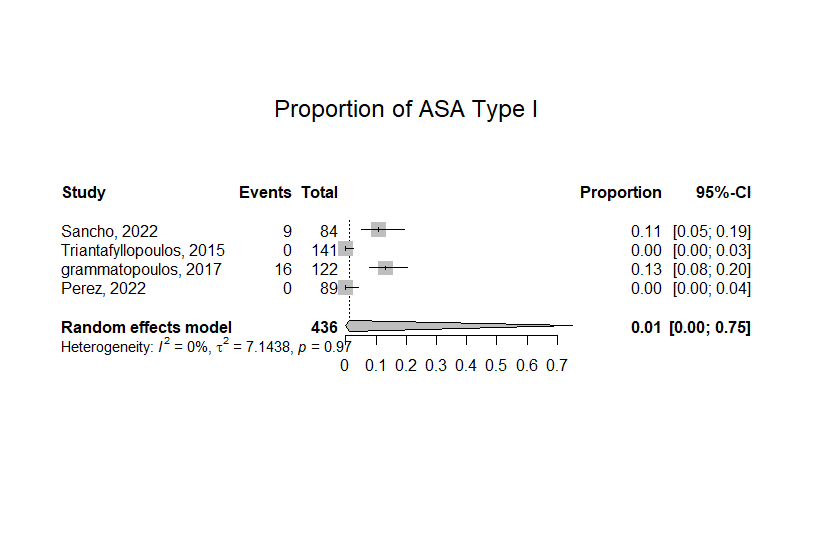

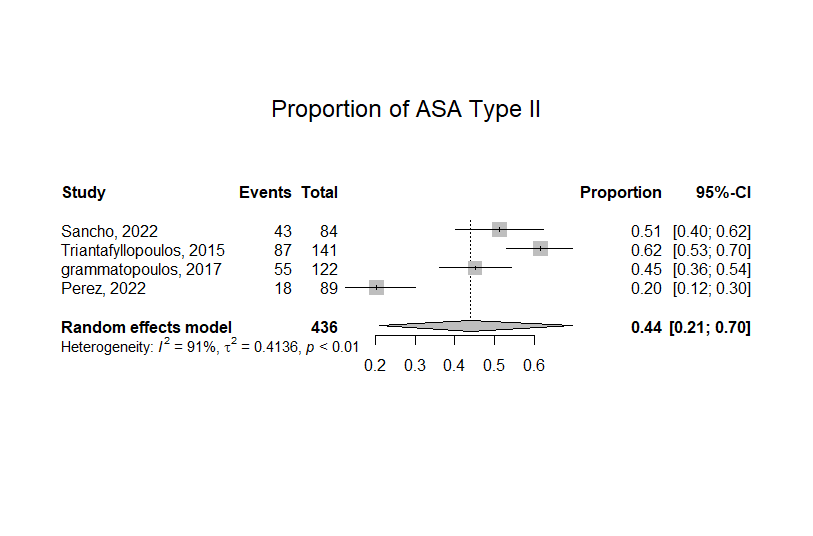

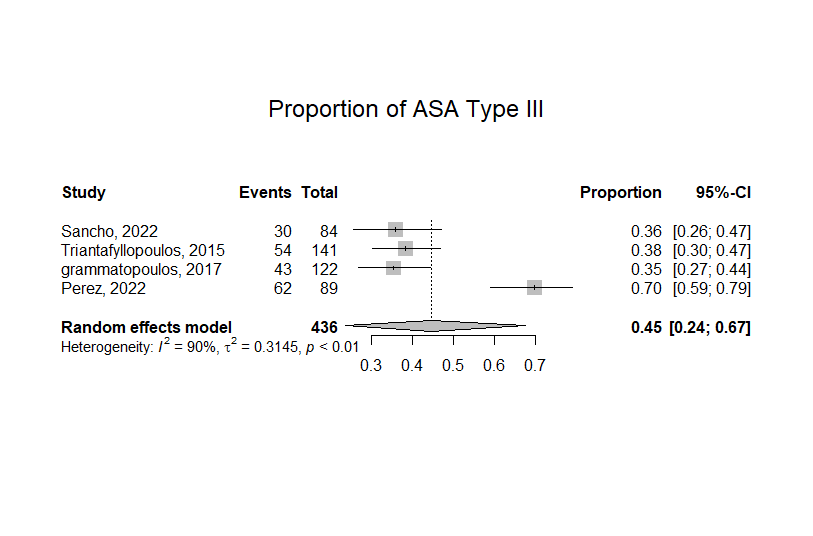

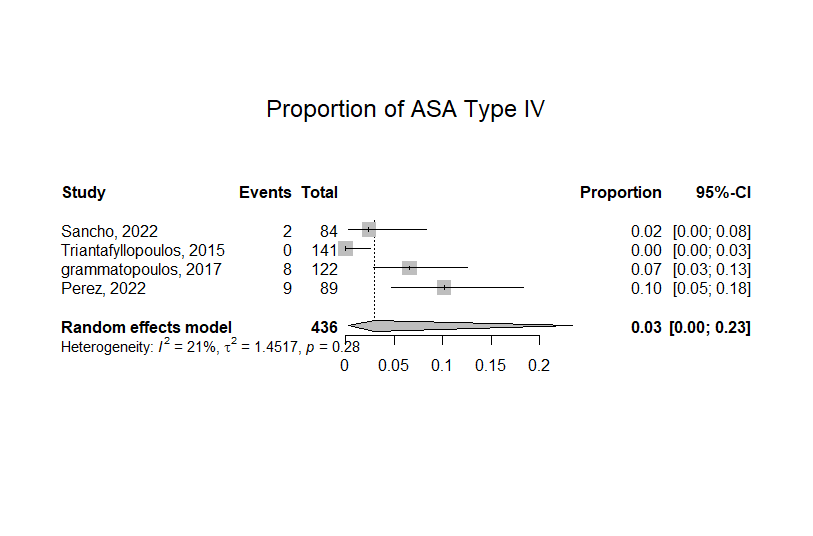

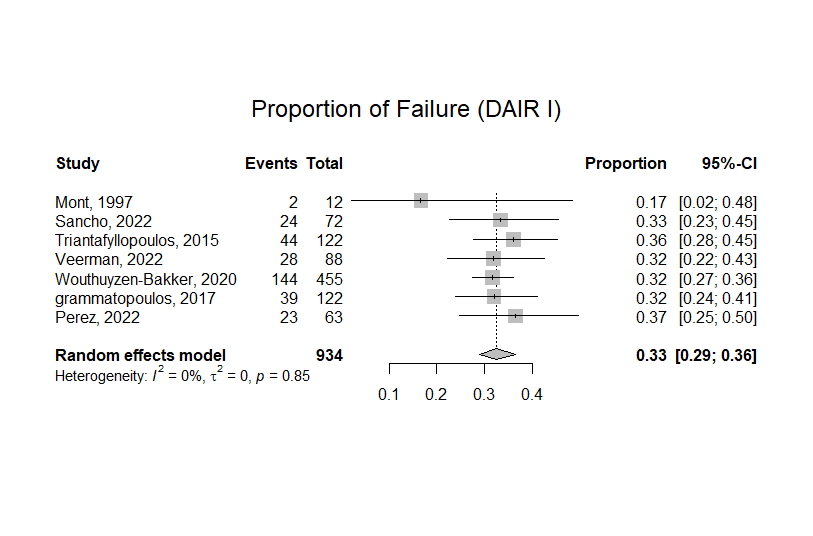

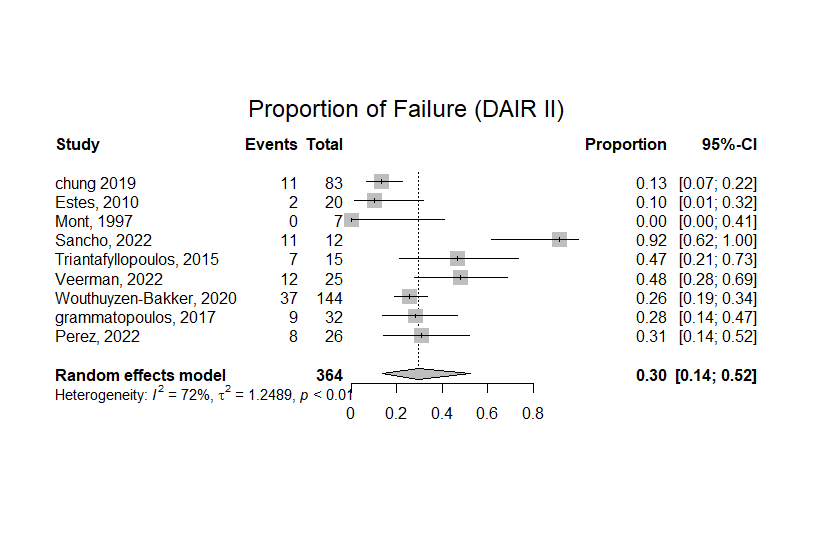

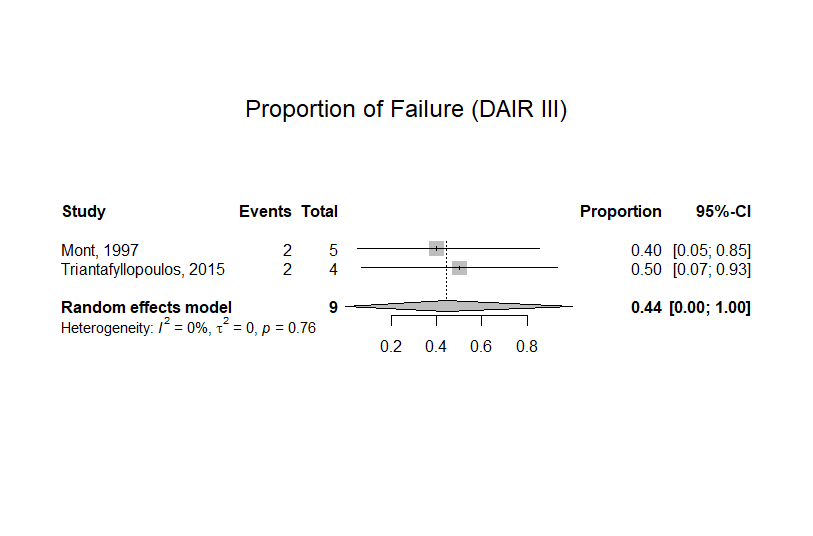

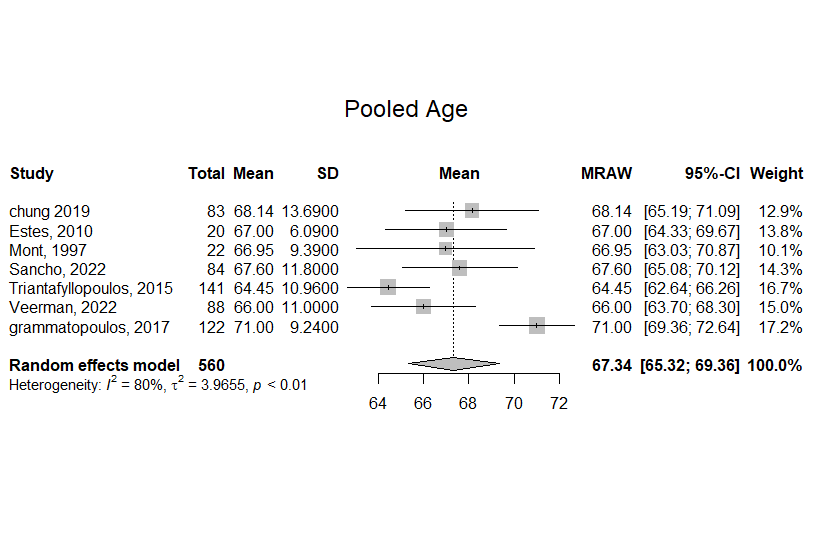

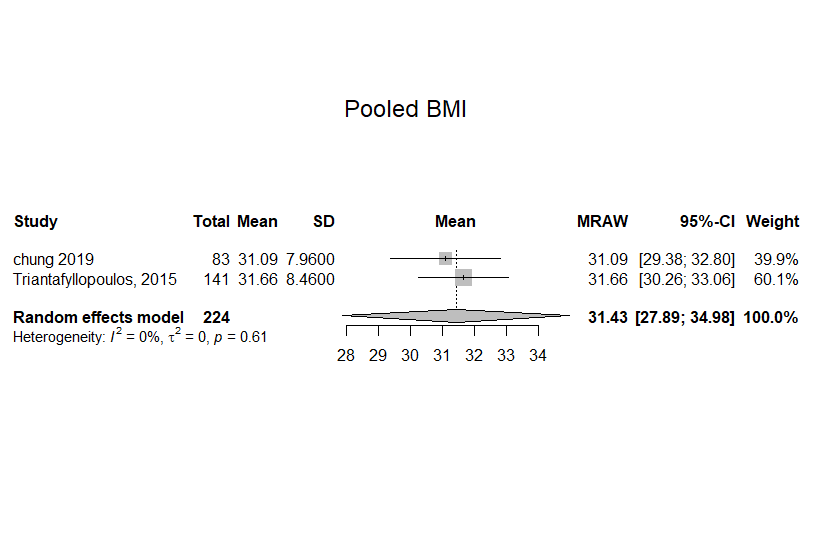

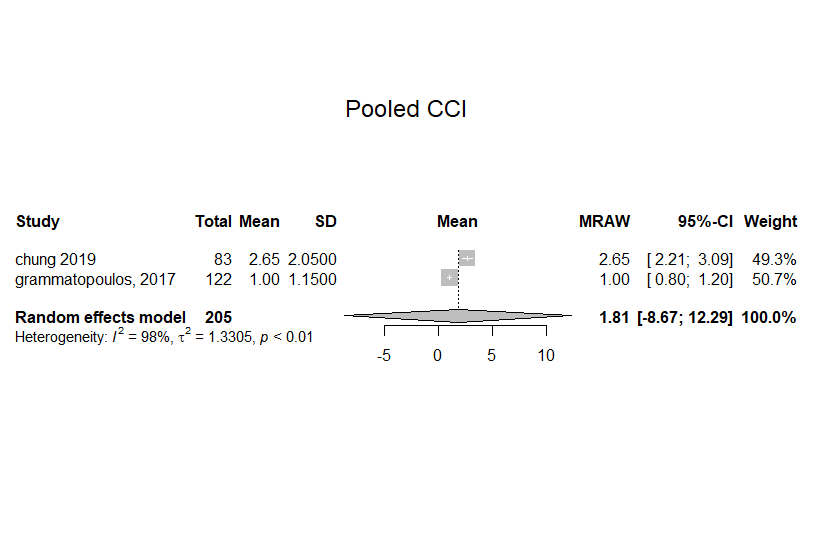

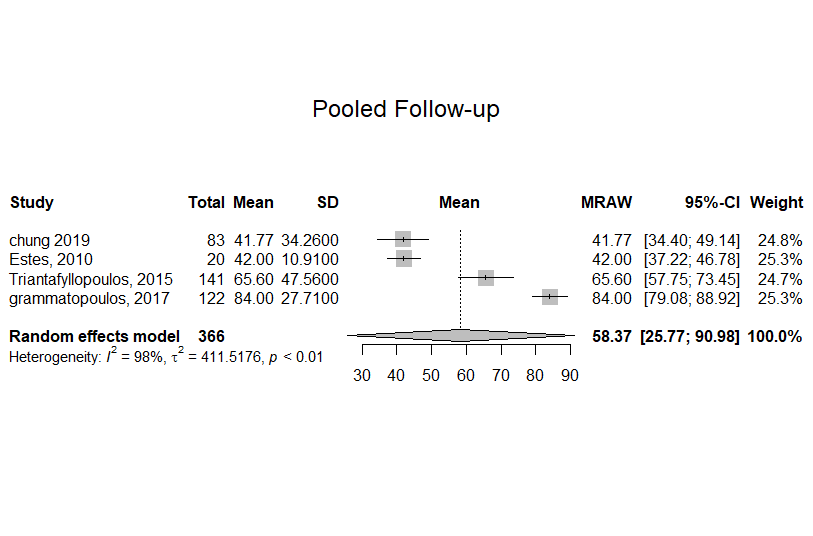

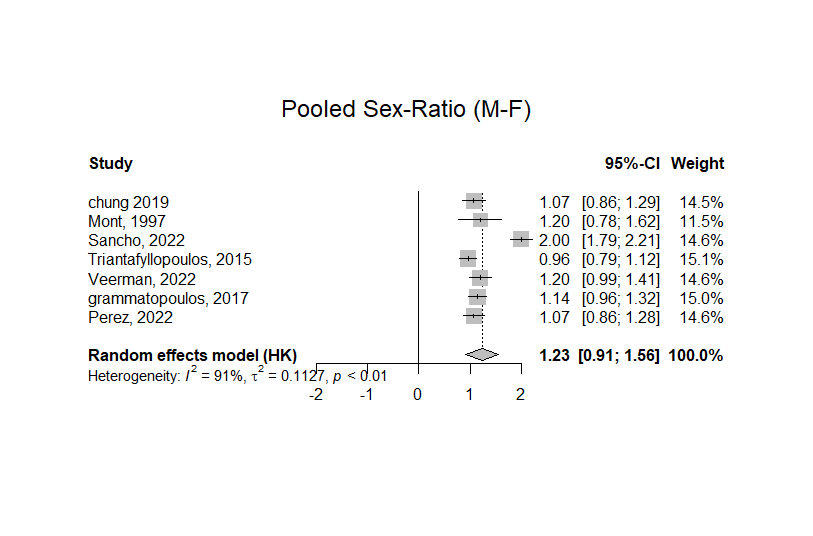

Supplement: Supplementary file 1 — Supplementary file1 (DOCX 38328 KB) [file 590_2024_4091_MOESM1_ESM.docx]
